# Navigating parenthood: a qualitative study of parental guidance and support in Norwegian child health clinics

**DOI:** 10.1080/17482631.2025.2605632

**Published:** 2025-12-21

**Authors:** Elise Tveråmo Aastveit, Bente Prytz Mjølstad

**Affiliations:** a General Practice Research Unit, Department of Public Health and Nursing, Faculty of Medicine and Health Sciences, Norwegian University of Science and Technology, NTNU, Trondheim, Norway

**Keywords:** Parental guidance, child health clinics, primary care, life course health development, public health nursing, adverse childhood experiences, general practice

## Abstract

**Background:**

Previous research shows that early childhood care has a significant impact on lifelong health. Supportive and nurturing parenting promotes resilience, while adverse childhood experiences (ACEs) increase the risk of health and developmental challenges. Studies also indicate that the foundation of the parental role is shaped by one’s own upbringing. In Norwegian antenatal care and child health clinic (CHC) services, healthcare professionals are expected to discuss parenting with parents. However, we have limited knowledge about how parental guidance is delivered and experienced during CHC consultations.

**Objective:**

To explore how parents and public health nurses (PHNs) experience the integration of parental guidance in routine CHC consultations, particularly in relation to challenges in early parenthood, and how these services can best support families through existing contact.

**Methods:**

We conducted a qualitative interview-based study consisting of in-depth interviews with eight mothers and one father, as well as two focus group interviews with eight PHNs from two medium-sized municipalities in Norway.

**Results:**

Based on an analysis of interviews with both parents and PHNs, four themes were developed: 1) challenges in parenthood: from time constraints to taboo emotions; 2) experiences from their own upbringing: continuation and change; 3) encountering the child health clinic: safe and accessible – primarily for mother and child; and 4) parental guidance: unnoticeably present.

**Conclusion:**

Parents and PHNs described CHCs as safe and accessible but noted that parental guidance was often subtle or unclear. PHNs emphasized joint reflection around parenthood but faced time constraints. Parents valued reflecting on their own upbringing and called for extended follow-up and greater inclusion of fathers. Tailoring guidance to family needs — combining reflective and direct approaches — may strengthen support and promote family relationships.

## Background

The way children are cared for in their earliest years shapes their development and lays the foundation for lifelong health. Nurturing and responsive caregiving fosters resilience, helping children cope with adverse life events, while a lack of such support may increase vulnerability (Boyce et al., 2021; Garner & Yogman, 2021; Schroeder et al., 2021; Shonkoff et al., 2021). This illustrates a life-course perspective, emphasising how health develops dynamically over time, and highlighting the importance of early prevention and health promotion (Halfon et al., 2014).

The groundbreaking 1998 Adverse Childhood Experience (ACE) study demonstrated a strong link between ACEs and mental and physical health problems in adulthood. A dose-response relationship was found, with more ACEs correlating with more health issues (Felitti et al., 1998). These findings have since been confirmed by numerous international studies (Hashemi et al., 2021; Tomasdottir et al., 2015; Wang et al., 2023; Winning et al., 2015).

While the original ACE study focused on adult health outcomes, recent research globally and in the Nordic region has also linked early adversity to long-term health and developmental challenges in children and adolescents. These include psychological difficulties such as emotional distress, social challenges like relational problems, and physical health issues such as obesity and overall morbidity and mortality (de Vries et al., 2025; Moriya et al., 2024; Rod et al., 2020; Schroeder et al., 2021). Norwegian studies, including the HUNT study, reveal that 4−7% of adults report having experienced a difficult childhood (Haugland et al., 2021; Tomasdottir et al., 2015). Among children in Norway, one in twenty is exposed to severe violence in their homes (Reneflot et al., 2019).

Parental roles are influenced by one’s own upbringing, and unprocessed ACEs and thought patterns can negatively impact parenting and predict problematic parent-child relationships (Carson, 2011; Murphy et al., 2014). Parental ACE scores are directly linked to emotional availability, discipline techniques, and children’s behavioural issues (Rowell & Neal-Barnett, 2022). Parents with ACEs may struggle to meet their children’s needs (Wattanatchariya et al., 2024), highlighting the need for early detection and intervention to enhance parenting quality (Murphy et al., 2014; Rowell & Neal-Barnett, 2022; Wattanatchariya et al., 2024).

The impact of ACEs can span multiple generations (Wang, 2022), particularly between mothers and children, by influencing children’s behaviour and health outcomes (Pereira et al., 2017; SmithBattle, 2018). Breaking the cycle of ACEs requires, among other factors, recognising their impact, fostering a strong parental desire for change, seeking support, and promoting resilience through communication, love, and family bonds (Woods-Jaeger et al., 2018). Good parenting and secure relationships are vital to a child’s upbringing, as children with secure attachments show better mental health, resilience, and self-esteem (Helsedirektoratet [Norwegian Directorate of Health], 2017; Horne et al., 2014).

While international research highlights the importance of early relational health and parenting support, Norway offers a unique context for universal early intervention. Early intervention and prevention are national public health priorities (Helleland et al., 2018; Horne et al., 2014), and healthcare professionals are expected to address parenting during both antenatal and postnatal care. All Norwegian women have access to free antenatal care with a midwife and general practitioner (GP), ensuring regular consultations, information, and emotional support (Helsedirektoratet [Norwegian Directorate of Health], 2022). After birth, families are included in the universal and free Norwegian child healthcare programme, which provides 14 standard consultations for children aged 0−5 years at child health clinics (CHCs) (Helsedirektoratet [Norwegian Directorate of Health], 2017). Most of these take place during the child’s first year, reflecting the programme’s strong emphasis on early intervention. Parents are legally required to ensure that their child receives these health consultations, with a participation rate of 95-100% (Glavin et al., 2023). Consultations are primarily conducted by public health nurses (PHNs) and include physical measurements, developmental milestones, and vaccinations. GPs attend four key consultations. Some consultations are group-based, allowing parents to share experiences and learn together (Helsedirektoratet [Norwegian Directorate of Health], 2017).

Programme guidelines emphasise that parents should be encouraged to discuss their concerns or questions during visits. They also recommend guidance on parent-child attachment, emotional support, and addressing parental mental health. The legal responsibility of the programme is to detect and prevent violence and abuse (Helsedirektoratet [Norwegian Directorate of Health], 2017). In recent years, CHCs have gradually shifted their focus from the mother-child relationship to the whole family with increased emphasis on including fathers (Høgmo et al., 2023; Solberg & Glavin, 2018).

Several qualitative studies have explored how parents and health professionals experience parenting challenges and support in primary care, highlighting parental insecurity, the value of relational continuity, and barriers to discussing sensitive topics (Berg et al., 2021; Robstad et al., 2022; Sundsbø, 2022). However, little is known about how parenting guidance is delivered in routine consultations and how it is experienced by parents.

A review has noted that the effects of general antenatal education on childbirth and parenthood remain largely unknown (Gagnon & Sandall, 2007). In Norway, several parental guidance programmes are available, but many lack high-quality research and evidence of long-term effectiveness (Helsedirektoratet [Norwegian Directorate of Health], 2017). Knowledge of their scope and implementation is also limited.

While parenting programs can reduce child maltreatment (Chen & Chan, 2016), and promote positive parenting (Bjørknes and Ortiz-Barreda, 2021), research on parents’ desire for guidance is limited (Bjørknes and Ortiz-Barreda, 2021). A 2021 parental evaluation of Norwegian CHCs found overall high satisfaction, yet many parents report difficulties obtaining advice on child-rearing (Kaiser et al., 2021). Furthermore, many first-time parents report the need for more prenatal and postnatal information and preparation for parenthood and newborn care (Ahldén et al., 2012; Entsieh & Hallström, 2016).

This study aimed to explore how parents and public health nurses (PHNs) experience the integration of parental guidance in routine CHC consultations, particularly in relation to challenges in early parenthood, and how these services can best support families through existing contact.

## Material and method

### Study design and setting

We conducted a qualitative study from August to October 2023 to explore parental guidance from the perspective of parents and PHNs at three CHCs in two medium-sized municipalities in Central Norway in a suburban setting. Interviews were conducted at the CHCs, participants' homes, or via Microsoft Teams.

### Recruitment and participants

PHNs were recruited through email invitations to the leaders of seven CHCs. Two of these leaders agreed to allow their PHNs to be invited to participate in the study. Each PHN provided informed consent prior to participation. A total of eight PHNs (all female, average age 46, average 12 years of experience—see Table I) took part in the study, divided into two focus groups.

**Table I.** Characteristics of the study participants; public health nurses (A-H), F = female, M = male.

Public health nurse	Child health clinic interview	Gender and age	Experience as a public health nurse (years)
A	1	F 55−60	27
B	1	F 40−45	10
C	1	F 30−35	3
D	1	F 35−40	5
E	1	F 40−45	9
F	2	F 55−60	22
G	2	F 45−50	7
H	2	F 45−50	10

Parents were recruited through flyers and in-person engagement at the same CHCs as the participating PHNs. Recruitment aimed to ensure variation in age, gender, and parenting experience. Each parent provided informed consent prior to participation. A total of nine parents (eight female, one male, average age 34.5, mostly with multiple children—see Table II). participated in individual interviews. Six of the parents worked in health or social services, while the others had varied occupational backgrounds.

**Table II:** Characteristics of the study participants; parents (1−9), F = female, M = male.

Parent	Gender and age	Marital status	Number of children	Age of children (years)	Cultural background other than Norwegian?
1	F 35−40	Cohabitant	3	0−8	No
2	F 30−35	Married	2	0−2	No
3	F 30−35	Cohabitant	2	0−5	No
4	F 30−35	Cohabitant	3	0−14	No
5	F 30−35	Married	1	0	Yes
6	F 40−45	Married	2	1−5	No
7	F 35−40	Married	3	0−6	No
8	F 30−35	Married	2	0−4	No
9	M 35−40	Married	4	1−11	No

### Data collection

We conducted in-depth interviews with parents to enable a more personal and detailed exploration of their experiences and thoughts. Focus groups with PHNs were chosen to facilitate dynamic discussions, using group interactions to generate a wider range of insights and ideas. The focus groups lasted 53−57 minutes, while the individual interviews ranged from 36 to 48 minutes. All interviews were recorded, stored in a secure storage area (NICE1), and transcribed by the first author (ETA). Anonymization of the data material was a key part of transcribing, particularly when potentially sensitive topics were discussed.

Interview guides were used for both focus groups and in-depth interviews, while also allowing for spontaneous exploration of participants’ thoughts (see supplement 1). The interviews explored how parenthood and parental guidance were addressed and prioritised at CHCs, the challenges participants faced in parenting and how CHCs could support them, and how parents’ childhood experiences influenced their parenting. Participants were also asked whether they found it appropriate to discuss such experiences at CHCs.

### Analysis

The data were analysed using reflexive thematic analysis (Braun & Clarke, 2006). This method identifies, analyses, and describes patterns and opinions within a dataset, aiming to organise and detail the data while also interpreting the key aspects of the research topic (Braun & Clarke, 2006, Braun & Clarke, 2023).

Thematic analysis was conducted by ETA, a medical student at the time of data collection and analysis, and BPM, an experienced qualitative researcher and GP with clinical experience from CHCs and with a research interest in health consequences of early adversity. During data collection, ETA observed the PHNs in their daily work, which contributed to her contextual understanding of the setting. Our different backgrounds informed the analysis and supported reflexivity throughout. We were aware of how our professional experiences and preunderstandings could influence interpretation, and we engaged in regular discussions to reflect on this.

The analysis was guided by the following research questions: How do parents experience their parental role and the guidance they receive from CHCs? How do PHNs understand and approach parental guidance? And in what ways do PHNs’ perspectives complement, contrast with, or contextualise parents’ experiences?

Following the six phases of Braun and Clarke’s reflexive thematic analysis (Braun & Clarke, 2006), we began by individually reading the parent interviews (*phase 1: familiarisation with data*). After this initial review, we read the interviews transversely, focusing particularly on parents’ experiences with the parental role, current parental guidance, and desired improvements (*phase 2: generating initial codes, and phase 3: searching for themes*). During the coding process, we generated both semantic codes, capturing explicit content, and latent codes interpreting underlying meanings. The codes were grouped into themes. The authors met regularly during this phase to discuss emerging impressions, explore different interpretations, and reflect on how our perspectives shaped the analysis. Differing thoughts and interpretations were discussed before agreeing on the final themes (*phase 4: reviewing and phase 5: defining, and naming themes*). The same phases were applied to the PHN focus group interviews with a focus on how PHNs understand and approach parental guidance. After analysing the parent interviews and the PHN focus group interviews separately, we then compared and integrated the themes to explore how the PHNs’ perspectives complemented, contrasted with, or contextualised the parents’ experiences. The final themes were presented with illustrative quotes from the parents, supplemented with insights (quotes) from PNHs, to enrich and contextualise the findings (*phase 6; producing the report*).

### Ethics approval and consent to participate

The study was conducted in accordance with the ethical principles of the Declaration of Helsinki, and the independent authority, the Norwegian Centre for Research Data (NSD), approved the study (Ref. nr 353798). The Regional Ethical Committee in Central Norway assessed the study, but no formal application was required (Ref. nr 601131), in accordance with Norwegian legislation and guidelines for health research. Ethical considerations were taken into account at all stages of the study, and written informed consent was obtained from all participants.

## Results

Through the analysis, we developed four themes reflecting the challenges of parenthood and the nature of parental guidance: 1) challenges in parenthood: from time constraints to taboo emotions; 2) experiences from their own upbringing: continuation and change; 3) encountering the child health clinic: safe and accessible—primarily for mother and child; and 4) parental guidance: unnoticeably present.

### Challenges in parenthood: from time constraints to taboo emotions

A recurring theme across the interviews was the everyday challenges of parenting. Nearly all parents described common difficulties such as time constraints, as well as more sensitive and taboo emotions. While some parents adapted easily to parenthood, others found it unexpectedly difficult and overwhelming. Their expectations seemed to shape their experience.

*I found it much more demanding and wish it were easier to express that openly in society. That having small children isn’t just rosy.* (Parent 2)

Both PHNs and parents cited societal influence and social media as sources of insecurity for some parents. All agreed that professional advice was most beneficial.

Some parents also described difficulty in managing their emotions and staying calm, especially when exhausted. Both parents and PHNs expressed that sleep deprivation could negatively affect mental health and trigger irritability or distress.

*I felt short-tempered and angry when I slept little, and it can be hard to talk about because you’re afraid people will think you’re not a good parent.* (Parent 2)

PHNs focused on parents’ well-being during the consultations. They also showed the video In Safe Hands (Original title:I trygge hender) in group sessions to address taboo topics, such as anger and violence against children. However, one parent felt that this would be better suited to one-on-one settings. Overall, many agree on the importance of discussing these topics.

*Standing in the middle of the night feeling like you want to throw your child out of the window. It’s okay to feel that way, but you need tools to handle it.* (PHN C)

All parents valued acknowledging their children's emotions without dismissal or divergence, and asked for more concrete advice about handling children’s emotions. Some parents and PHNs highlighted the necessity of setting clear boundaries.

*It’s becoming harder for parents to handle boundary-setting, as the child should decide more for themselves. This seems to be more of a challenge now than in the past. Perhaps there should be more guidance around that?* (PHN E)

PHNs also noted a marked shift in parenting between ages 2−4, as children become more independent and defiant. Despite this, there were no scheduled consultations during this period:

*You receive a lot of useful information about the child at the child health clinics, but two years after birth is when you might feel like you need more tools in the parental role — at least for my part.* (Parent 9)

Several parents reported that their most significant challenges arose after the first year and expressed a need for more guidance and professional support during this phase.

### Experiences from their own upbringing: continuation and change

When invited to reflect on their own childhood and what they would carry forward as parents, all participating parents acknowledged that their upbringing influenced their parenting, whether consciously or unconsciously. Most highlighted positive experiences they hoped to pass on, including family traditions, shared meals, housing routines, vacations, and core values. A common goal was to create a safe, supportive, and stable family environment.

*What you want is security. It’s what I grew up with too — very much a nuclear family. (…) So, it's important to me as well that my kids know they can always come to me, no matter what.* (Parent 4)

At the same time, many parents expressed a desire to parent differently from their own parents, particularly in terms of emotional openness and communication. They described wanting closer relationships with their children and being attuned to their thoughts and feelings, reflecting a broader societal shift.

*What I reflect on is that I don’t want to do things like my parents did. (laughs a bit) I want us to express our feelings (*…*) You shouldn’t be scolded if you make a mistake. We need to try to understand and learn from it.* (Parent 7)

Some parents also reflected on the more difficult aspects of their own or their partner’s upbringing, such as divorce, anger, financial hardship, and substance misuse. These experiences often foster self-awareness and a conscious effort to break negative patterns and parent more intentionally.

PHNs emphasised that reflecting on one’s childhood is key to raising awareness and fostering changes in parenting practices.

*It may not always be easy for them [parents] to understand. Even if they’ve been told or have talked about how current experiences may be connected to their childhood, it can be quite complex to work through. So we often need to walk alongside them in that process.* (PHN E)

However, parents reported that such reflections rarely came up during consultations with the PHN. While opinions varied on the necessity of discussing the topic, some emphasised its importance:

*Having a small checkpoint regarding your upbringing is really important (…) It can catch a lot. You become a bit restrictive about what you want to share, because you fear the involvement of child protective services. But I think it’s important to create more openness and security to share.* (Parent 3)

Overall, all parents agreed that discussing their upbringing was acceptable when approached in a safe and respectful manner.

### Encountering the child health clinic: safe and accessible — primarily for mother and child

In general, all parents expressed satisfaction with the services provided at the CHCs. They felt well cared for and appreciated the support they received. Both parents and PHNs described the CHCs as safe, supportive, and accessible. Several parents emphasised specific qualities that contributed to this sense of safety, such as the openness of the CHCs, the ease of initiating contact, and the availability of help at short notice, even outside scheduled appointments. These aspects fostered trust and made it easier to seek help when needed.

*If anything comes up, you can get in touch. They help on short notice. I feel there's a very open dialogue and a low-threshold. You can talk about anything. And the fact that it's free makes it easier to reach out when you need to.* (Parent 8)

The PHNs also pointed out that the CHCs were non-stigmatising, as virtually all families participated regardless of background. One PHN described the CHCs as *“part of the circle of security”,* with the PHN being a safe haven for parents.

*We have the Circle of Security — where parents are the secure base for their children. But sometimes we feel that we are the secure base for the parents* *— the child health clinic.* (PHN A)

Despite the sense of safety, parents and PHNs noted that mothers primarily attended CHCs. While fathers were invited, some described the consultations as being focused on mothers. Everyone agreed on the importance of addressing the father’s role in gender equality and family well-being.

*It should be a bigger focus. I think fathers are often forgotten. I agree with the priorities [of mother and child], but there could be room to include more time for the father as well.* (Parent 9)

*It is very important to involve the partner during home visits. Perhaps also provide information about the CHC’s services during pregnancy follow-up. Regularly signal that we are not a mother–child clinic; we are for the whole family, including fathers and partners.* (PHN C)

To include fathers at the CHCs better, both PHNs and parents suggested introducing dedicated father consultations and group sessions, along with more open discussions about fatherhood in general. One mother emphasised the value of male-only spaces for sharing experiences:

*Separate groups for fathers are a great idea. I think it’s a different threshold for opening up in a group with other men, compared to when the mothers are there. (…) What are they feeling? Because it's surely different from what I'm feeling.* (Parent 4)

Furthermore, the interviewed father also expressed interest in such initiatives aimed at increasing father involvement at CHCs.

### Parental guidance: unnoticeably present

Many parents struggled to recall whether they had received parental guidance or the extent to which it had been offered. Some participants noted that they did not explicitly express the need for such support. This ambiguity surrounding the availability and visibility of parental guidance left some parents feeling underprepared for the emotional and relational aspects of parenting. One parent illustrated this gap through a striking comparison:

*When people get a dog, they sign up for dog training classes a year in advance. But when you become a parent, the focus is mostly on birth and the practical stuff. At least for me, I didn’t pick up many tools for being a parent, not when it comes to the emotional side of becoming a parent, on things like parenting and upbringing.* (Parent 9)

Several parents expressed a desire for a clearer and more structured approach to parental guidance, including concrete questions about common challenges and more openness around difficult and taboo topics.

*People usually avoid bringing up what’s really painful or when things aren’t going the way they should. (…) The child health clinics should take more responsibility for this, by saying, 'Today, we’re going to discuss this topic.' (…) They shouldn’t be afraid to ask the tough questions.* (Parent 8)

In addition, some parents, particularly first-time parents, expressed a need for more direct and practical advice rather than reflective questioning:

*If I bring a problem to my public health nurse, I don't want to hear; 'What do you think?' I want concrete advice on what I can try.* (Parent 3)

Parents who recalled receiving parental guidance described it as being open and parent-focused. Most valued the opportunity to discuss their own topics and felt comfortable doing so. The PHNs confirmed the prioritisation of parents’ concerns.

*I think that’s probably the most important part of the programme: What do they [the parents] want to address? Are they facing any challenges?* (PHN E)

PHNs in both focus groups emphasised that parental guidance begins during pregnancy and continues after birth, with a consistent focus on fostering parent-child interactions. They describe this guidance as being subtly embedded in everyday encounters, sometimes starting with observations made as early as in the waiting room.

*Parental guidance is integrated into everything we do; from breastfeeding advice to helping parents understand their child’s signals and eventually language.* (PHN C)

Because the guidance is integrated into routine care and often delivered informally, through conversations, observations, and practical advice, it may not always be perceived by parents as structured or explicit. Two of the interviewed mothers, both without professional backgrounds in health and social services, expressed they were unaware that CHCs offer parental guidance:

*Weight, height, and vaccine follow-up, but not the parental role. (…) I haven’t really thought of the child health clinics as having a role in that.* (Parent 7)

Furthermore, the PHNs described balancing many responsibilities in a limited time and noted variations in parental guidance across CHCs due to differing resources. They focused on reassuring parents and normalising the process of trial and error but pointed out a difficult balance between normalising and trivialising. They also encouraged parents to engage in independent reflection, an approach that contrasted with some parents’ wishes for more concrete and practical advice. As one PHN explained:

*There aren’t many definitive answers here — and some people appreciate that, while others don’t.* (PHN F)

Still, the PHNs acknowledged that more direct communication is necessary at times:

*Sometimes I think we need to learn to be a bit clearer - to communicate what we observe and what we're worried about.* (PHN C)

Taken together, the quotes illustrate that parental guidance is often integrated and informal, which can make it less visible to parents and that effective communication may require both reflective and direct approaches.

## Discussion

### Main findings

This study provides new insights into how parental guidance is integrated into routine CHC consultations and how parents and PHNs perceive its role in supporting early parenthood. While parents generally expressed satisfaction with CHC services, describing them as safe, accessible and open, the results also reveal a tension between perceived openness and the need for more direct support. Both parents and PHNs noted that parental guidance was often subtle or indirect. PHNs balance parental guidance with other tasks during consultations, often using reflective approaches rather than explicit advice. Some parents appreciated this style, while others preferred clearer and more direct communication, particularly regarding sensitive topics. The limited inclusion of fathers and the subtle nature of guidance may contribute to why some parents experienced the support as less visible. These findings should be understood in the context of structural and time constraints that limit PHNs’ opportunities to offer tailored guidance. The findings also highlight that the transition to parenthood is often more demanding than expected and that parents’ own upbringing shapes their approach to raising children. Together these findings raise questions about how parental guidance is framed and whether current practices fully meet the diverse needs of families.

To better understand these findings, we draw on previous research on parental experiences and CHCs and briefly incorporate theoretical perspectives such as the life course health development model (Halfon et al., 2014) and frameworks on adversity and resilience. These perspectives help illuminate how early life experiences and social expectations shape both parents’ needs and PHNs’ approaches to support.

### Challenges in parenthood: from time constraints to taboo emotions

Parents and PHNs described the challenges in transitioning to parenthood, with unmet expectations, societal pressures, and social media adding stress. According to a UK study, some mothers reported feelings of guilt due to unrealistic ideals, while online support was helpful for some but stressful for others (Finlayson et al., 2020). Providing realistic knowledge and preparation can ease this transition, as suggested by previous research (Entsieh & Hallström, 2016).

Parents reported mixed emotions in early parenthood, with sleep deprivation contributing to frustration, which was often difficult to discuss at child health clinics. Previous studies have shown that postpartum emotions fluctuate and are worsened by exhaustion (Finlayson et al., 2020), and that sleep-deprived mothers experience unsatisfactory support from PHNs (Pettersen Sandtrø et al., 2023). Our findings echo these challenges and underscore the need for open dialogue and tailored support.

PHNs and parents discussed “*I Trygge Hender”*, a video on parental anger and child abuse, shown in group consultations. However, one parent felt that these topics were better suited to the individual sessions. Discussing such topics requires trust, communication, and interpersonal skills from nurses (Kim & Choi, 2025). Additionally, the research advises a safe, private, and confidential setting to facilitate the disclosure of abuse (Heron & Eisma, 2021).

In addition, parents wanted more guidance on emotion regulation, boundaries, and defiance after the age of two. Research shows that clear yet balanced boundaries reduce youth deviance (Lassi, 2025) and that parents’ emotion regulation shapes toddlers’, suggesting that parent-focused interventions may enhance toddler development (Edvoll et al., 2023). Overall, our findings reinforce existing knowledge about the emotional and relational demands of early parenthood, while highlighting specific areas where CHCs may strengthen their support, particularly in addressing taboo topics and promoting emotional competence.

### Experiences from their own upbringing: continuation and change

Parents felt that their upbringing shaped their parenting style, leaning toward authoritarian tendencies. Previous research has linked authoritative parenting to well-being, while authoritarian or neglectful parenting leads to poorer outcomes (Blaasvær & Ames, 2019). Warmth and support promote positive development (Blaasvær & Ames, 2019) and secure attachment (Helsedirektoratet [Norwegian Directorate of Health], 2017).

Several parents in our study emphasised a desire to break negative cycles from their own childhoods, some viewing guidance from PHNs as essential in this process. Research emphasises the value of processing trauma, increasing ACE awareness, providing mental health support, and offering early intervention. Supportive communities and building resilience through love and communication are also emphasised as key strategies for preventing and replacing harmful parenting (Carson, 2011; SmithBattle, 2018; Woods-Jaeger et al., 2018). This aligns with the life course health development perspective (Halfon et al., 2014), which emphasises how early life experiences shape long-term health trajectories and the importance of timely, supportive interventions.

Although parents were generally open to discussing their upbringings, opinions on their necessity varied. U.S. studies have shown that ACE screening during pregnancy or during paediatric visits can help identify health risks and enable early trauma-focused support (Murphy et al., 2014; Olsen, 2018; Rowell & Neal-Barnett, 2022). Furthermore, low-threshold support has been shown to reduce the intergenerational transmission of mental health challenges by up to 40% (Bütikofer et al., 2024). These findings suggest that child health clinics may support parents in reflecting on and reshaping parenting practices, particularly informed by perspectives on adversity, resilience and life course development.

### Encountering the child health clinic: safe and accessible—primarily for mother and child

Parents were generally satisfied with child health clinic services, aligning with a 2021 Norwegian evaluation reporting 95% satisfaction and 91% receiving the support they needed (Kaiser et al., 2021). High satisfaction is likely reflected in the programme’s 95-100% participation rate (Glavin et al., 2023), which is also influenced by its mandatory nature. Parents and PHNs noted that mothers and children were the main attendees at CHCs. Similarly, a Swedish study found that while 70% of fathers visited the CHC at least once, only 38% attended regularly or quite often, and just 28% had participated in a parents’ group (Hallberg et al., 2010).

Several parents and PHNs in our study suggested dedicated father groups or consultations to increase fathers' involvement. While such initiatives have been attempted in some CHCs, participation remains undocumented and unevaluated (Solberg & Glavin, 2018). In parts of Norway, trial father groups led to strengthened networks, and 6 out of 10 fathers reported stronger bonds with their newborns (Killen, 2013).

Parents and PHNs also called for greater emphasis and open discussion on fatherhood. This need is supported by Solberg et al., who found that many fathers feel secondary and uncertain at CHCs yet wish for a more active postnatal role (Solberg & Glavin, 2018) and greater recognition as fathers, individuals, and equal partners in parenthood (Solberg et al., 2022). Complementing these findings, a UK study highlighted the importance of addressing fathers' mental health during pregnancy to benefit families and reduce future healthcare costs (Kowlessar et al., 2015). Recent studies (Salemonsen et al., 2022; Salemonsen et al., 2023) further show that fathers are concerned about their children’s future health and want to be more involved. Together, these findings underscore a desire for more inclusive practices at CHCs, where fathers are actively supported and acknowledged as equal caregivers.

### Parental guidance: unnoticeably present

Parents, PHNs, and guidelines all emphasise user participation. This emphasis is reflected in a Norwegian survey, where 64% of parents felt that they had a strong influence on the support they received, 25% to some extent, and 11% not at all, indicating the potential for increased involvement (Kaiser et al., 2021).

Several parents in our study struggled to recall discussions about their parental role during consultations and wanted to focus more on it. This aligns with a Norwegian qualitative study in which parents noted that the 2-year consultation prioritised physical and developmental aspects over mental health, parental guidance, and parent-child interaction (Mathiesen, 2016). Similarly, a Swedish review found that antenatal classes lacked information on parenting skills, emotional health, and relationships, leaving parents unprepared, and suggested early postnatal parenthood education (Entsieh & Hallström, 2016).

Although many parents appreciated the open and welcoming approach at child health clinics, they found it difficult to independently raise sensitive topics. According to a national survey, only 54% of parents sought child-rearing advice at CHCs despite high satisfaction with other follow-ups, suggesting that trust in nurses was not a concern (Kaiser et al., 2021). Our findings indicate that parental guidance may be perceived as “invisible” and that indirect approaches may not always meet parents’ expectations.

Parents in our study preferred direct questions on challenges and sensitive topics, aligning with studies showing that directly asking about abuse may facilitate victims' disclosure (Heron & Eisma, 2021). Furthermore, Harder et al. found that in general, effective nurse-parent communication can be authoritative or guiding, depending on parents’ needs (Harder et al., 2024). However, misjudging these needs or offering unsolicited advice can hinder support (Eronen et al., 2010; Harder et al., 2024).

PHNs in our study cited heavy workloads and varying resources across CHCs as challenges, echoing previous Norwegian research describing an evolving PHN role with time-consuming responsibilities and insufficient resources (Dahl, 2018). The PHNs prioritised reassuring parents and promoting independent problem-solving. Similarly, an Australian study found that supporting parental decision-making and providing resources was more effective than expert advice (Eronen et al., 2010). Taken together, these findings reveal a gap between the intention to support and parents’ experience of guidance. Structural constraints, such as limited time and competing responsibility, may further reduce opportunities for tailored support. By identifying how indirect communication and structural constraints may limit the visibility and impact of parental support, our study points to a need for clearer, more responsive approaches in CHCs.

### Methodological strengths and limitations

A key strength of this study is the triangulation of data from both parents and PHNs, as well as the diversity in participants’ backgrounds. The study included PNHs from three CHCs in two different medium-sized suburban municipalities in Central Norway, with varying age and experience levels. PHNs’ collegial relationships facilitated an open and secure discussion climate. The findings are most transferable to medium-sized suburban municipalities in Norway, as services may differ between smaller and larger municipalities, as well as in more urban or rural areas. However, the insights are likely relevant nationally and internationally to child health clinic services in similar settings.

Recruiting CHCs was challenging; only three of the seven invited clinics participated because of time constraints. Similarly, recruiting parents, particularly fathers, proved difficult, resulting in a sample that primarily reflected mothers’ perspectives. Only one father participated, which limits the study’s ability to explore fathers experiences. Most parents had multiple children, providing insights into both first-time parenting and evolving security over time. The study included diverse childhood experiences but lacked representation of single parents and same-sex couples. Additionally, six of the eight mothers worked in health or social services and their familiarity and experiences may have led them to reflect differently than parents without such experience. While parents were recruited from the same CHCs, we do not know whether they knew each other. Shared local context may nonetheless have influenced their experiences and perspectives.

Despite these limitations, the study demonstrates strong informational power through triangulation of data sources, participant diversity, and depth and richness of the insights generated (Malterud et al., 2016).

## Conclusion

This study provides insight into how parents and PHNs experience the follow-up programme at child health clinics. Parents describe the service as safe, open and accessible, appreciating the availability and joint reflection. At the same time, both parents and PHNs noted that parental guidance was often subtle or indirect. While some parents valued this style, others found it difficult to interpret or act upon, especially when facing sensitive challenges. PHNs emphasised the importance of promoting independent problem-solving but also pointed to time constraints and heavy workloads as barriers to more tailored support.

Many parents valued reflecting on their own upbringing and called for extended follow-up beyond the child’s first year, particularly between the ages of two and four. Both parents and PHNs emphasised the need for greater father involvement. Tailoring guidance to family needs, combining reflective and direct approaches, may enhance support and contribute to strengthening family relationships. These findings suggest practical implications for public health nursing, including the use of reflective and direct questions, structured follow-up in toddler years, and father-inclusive strategies. Future research should explore how such approaches can be implemented effectively in everyday practice.

## Supplementary Material

Supplementary material Supplementary 1

## Data Availability

On reasonable request, the datasets will be made available.
