# Supplementary material for: Navigating parenthood: a qualitative study of parental guidance and support in Norwegian child health clinics
Source: Int J Qual Stud Health Well-being. 2025 Dec 21;21(1):2605632. doi: 10.1080/17482631.2025.2605632 (PMC12720690; doi:10.1080/17482631.2025.2605632)
Supplement: Supplementary material — Supplementary 1 [file ZQHW_A_2605632_SM0094.docx]

Supplement 1:

## Interview Guide for Parents of Young Children

**Participant characteristics:** Age, gender, education, first-time or experienced parent, relationship or single caregiver, ethnicity? Other?

**Introduction read at the start:**

Becoming a parent is, for many, one of life’s most important events and most parents want the best for their children. When preparing for parenthood, it is natural to reflect on one’s own upbringing, how it was, and how those experiences shaped one’s own parenting role.

The child health clinic program encourages health personnel to talk to parents about their parenting roles. This study explores **to what extent this is done—and how**. We are interested in what parents themselves think about the challenges of parenting and how the child health clinic can best support them in the contact they already have with the service.

**Part 1:**

**Can you start by telling us about your experience with the child health clinic regarding this topic?**

- To what extent have you experienced this being addressed? Is it prioritized?
- What do you think about the way it is done?
- To what extent have you felt able to raise concerns about parenthood with health personnel at the clinic?
- To what extent do you feel there is a family focus that includes both partners at the clinic?
- How important do you think it is to focus on your father’s role? What could help fathers take a more active and inclusive role?
- What other sources of parenting guidance have you used?

**As a parent, what do you think are the challenges in the parenting role?**

- What expectations did you have for parenthood before birth? How have they evolved?
- How did you experience the transition to parenthood?
- What kind of parent do you want to be?
- How do you handle your own emotions as a parent?

**What topics do you think are most important to discuss regarding the parenting role?**
(e.g., interaction, parenting style – discipline, nutrition – eating habits, sleep, setting boundaries, siblings’ roles)

- Is there a specific topic you think is especially important?

**How can the child health clinic best support parents in their parenting role, through the contact they already have with the service? Any other thoughts…?**

**Part 2:**

When preparing for parenthood, it is natural to think about your own upbringing and how it was, and how your own parents handled things.

- What significance do you think your own childhood experiences have had in your parenting role?
- Have you reflected on what you want and do not want to carry forward from your upbringing into your parenting?
- To what extent do you think it would be okay to discuss these kinds of topics with nurses at a child health clinic?
- Anything else you’d like to add?

## Interview Guide for Public Health Nurses

**Participant characteristics:** Age, gender, work experience, size of child health clinic/municipality, and collaborating partners at the clinic.

**Introduction read at the start:**

Becoming a parent is, for many, one of life’s most important events and most parents want the best for their children. When preparing for parenthood, it is natural to reflect on one’s own upbringing, how it was, and how those experiences shaped one’s own parenting role.

The child health clinic program encourages health personnel to talk to parents about their parenting roles. This study explores the extent to which this is done and how. We are interested in what you as public health nurses think about the challenges in parenting and how the clinic can best support parents in the contact they already have with the service.

**Can you start by sharing your own experiences from working at the child health clinic on this topic?**
(Feel-free to share anonymized case examples)

- What do you think are the biggest challenges in the parenting role?
- What are the most important topics to discuss regarding parenting?

There were many topics to be covered during the consultations. To what extent do you feel that your parenting role is prioritized?

**Can you speak a bit about the training and competence among public health nurses in addressing the parenting role at the clinic?**

- Was this a key part of your education? Continuing education/courses?

Do you think parenting guidance is practiced differently across child health clinics? How?

- To what extent do you feel there is a family focus that includes both partners at the clinic?
- How important do you think it is to focus on your father’s role? What could help fathers take a more active and inclusive role?

Traumatic childhood experiences can impact one’s functioning as a parent if not processed.

- To what extent do you discuss with your parents how their own upbringing might influence their parenting role?
- To what extent do you feel it is appropriate to discuss such topics with the clinics’s users?

**General:**

- What works well regarding parenting guidance today?
- What could be improved?
- Is there anything that parents express that they miss in child health clinic follow-up related to the transition to parenthood?
